# Randomised controlled trials and changing public health practice

**DOI:** 10.1186/s12889-017-4287-7

**Published:** 2017-05-30

**Authors:** Anne Cockcroft

**Affiliations:** 1 CIET Trust, Gaborone, Botswana; 2 Department of Family Medicine, McGill University, Montreal, Canada

## Abstract

One reason for doing randomised controlled trials (RCTs) is that experiments can be convincing. Early epidemiological experimenters, such as Jenner and the smallpox vaccine and Snow and his famous Broad Street pump handle, already knew the answer they were demonstrating; they used the experiments as knowledge translation devices to convince others.

More sophisticated modern experiments include cluster randomised controlled trials (CRCTs) for experiments in the public health setting. The knowledge translation value remains: RCTs and CRCTs can potentially stimulate changes of practice among stakeholders. Capitalising on the knowledge translation value of RCTs requires more than the standard reporting of trials. Those who are convinced by a trial and want to act, need to know how the trial relates to their own context, what contributed to success, and what might make it even more effective. Implementation research unpacks the back-story, examining how and why an intervention worked.

The *Camino Verde* trial of community mobilisation for control of dengue reported a significant impact on entomological indices of the *Aedes aegypti* vector, and on serological dengue virus infection and self-reported dengue cases. This important study should lead to studies of similar interventions in other contexts, and ultimately to changes in dengue control practices. This supplement is the back-story of the trial, providing information to help researchers and planners to make use of the trial findings.

Background articles include the full protocol, a systematic review of CRCTs of approaches for *Aedes aegypti* control, epidemiological and entomological findings from the baseline survey, and how baseline findings were used to set up the intervention. Secondary analyses of the entomological findings examine associations with the use of the larvicide temephos, and the impact of the intervention in different conditions of water supply and seasons.

Other articles describe implementation and other impacts: the underlying approach; implementation in the trial’s different social contexts; the different impact in women and men; the effects of using fish for vector control; the impact on household costs of personal protection and of cases of dengue illness; and ethical issues.

We hope this supplement will increase the knowledge translation value of the *Camino Verde* trial.

## Article

An important reason for doing randomised controlled trials (RCTs), even when there is already evidence in favour of an intervention, is that experiments can be very convincing. Epidemiology is built on a history of convincing experiments, real or contrived, such as Jenner and the smallpox vaccine [1], Snow and his famous Broad Street pump handle [2], and Semmelweis and postnatal sepsis [3]. In each case, the experimenter already *knew* the answer he wanted to demonstrate and used the experiment to prove the point to others; like many since, those early experiments were very effective knowledge translation devices.

Of course, the scientific discipline behind epidemiological experiments has grown hugely since the demonstrations of Jenner, Snow and Semmelweis, not least in requiring a comparison with a suitable, randomly allocated non-treated control group [4, 5]. Cluster randomised controlled trials (CRCTs) increase the scope for experiments in the public health setting. But the knowledge translation aspect remains: experiments still help to convince stakeholders by drawing them into a different reality, promising them a clearer view of the contrast between exposure and non-exposure. Experiments in the form of RCTs and CRCTs can be an important stimulus for changes of practice among individuals and communities, service providers, or decision makers.

Viewing RCTs *as* knowledge translation influences how we plan, implement and report on these experiments. To capitalise on the knowledge translation value of RCTs we need much more than the standard reporting of a trial offers. Those who are convinced, based on the RCT outcome, that an intervention can work and want to act on this conviction, need to know how the trial relates to their own context, what contributed to the success of the intervention, and what might make it even more effective. This is the realm of implementation research, which unpacks the back-story - what was done and what was measured - to examine how and why an intervention worked [6].

This supplement of BMC Public Health is the back-story, implementation research and secondary analysis, of the *Camino Verde* trial of community mobilisation for control of dengue in Mexico and Nicaragua. The British Medical Journal published the main methods and results of the trial [7] that demonstrated a significant benefit of adding community mobilisation to the existing government dengue control programme, which in both countries is based largely on the larvicide temephos. Previous trials of community mobilisation showed reductions in entomological indices of the *Aedes aegypti* mosquito, a key vector for dengue, as well as chikungunya, yellow fever and zika [8, 9]. But this was the first trial to show an impact on dengue virus infection (measured serologically) and on self-reported dengue cases [7].

So the *Camino Verde* trial is an important study that will hopefully lead to more studies of similar interventions in other contexts, and ultimately to changes in international practice for dengue control. The articles in this supplement provide additional information that researchers and planners might need in order to make best use of the trial findings.

Five articles describe the background for the trial in Nicaragua and Mexico. Andersson et al. describe the initial feasibility study undertaken in Nicaragua and present the full protocol of the trial [10]. A systematic review of CRCTs of different approaches for control of *Aedes aegypti*, updated to 2016, concludes that the evidence for community mobilisation approaches is stronger than that for chemical control measures [11]. Two articles describe epidemiological and entomological findings from the 2010 baseline survey of the trial in Mexico, which were used in setting up the intervention in Mexico [12, 13]; one article from Nicaragua describes the process of discussing findings from the initial feasibility study to co-design the trial intervention in Managua [14].

Entomological surveys during and at the end of the trial provided data for supplementary analyses to increase understanding of the vector indices in different circumstances. Two articles examine the use of temephos as a way of controlling mosquito breeding: one examines the lack of association between presence of temephos and vector indices in Managua [15], and another reports low coverage of the routine programme of temephos application and a reduction in temephos presence associated with the intervention in Mexico [16]. Two articles describe entomological indices in different conditions of season and water supply and examine the impact of the trial intervention in these different circumstances [17, 18].

Five articles consider implementation of the *Camino Verde* intervention, beginning with an explanation of the underlying approach of socialising evidence for participatory action (SEPA) [19]. The intervention implementation was quite different in Managua, Nicaragua, where there was already considerable community cohesion and collaborative action [20], and in the coastal regions of Guerrero state, Mexico, where conflict and insecurity had eroded community trust and social capital [21]. In both countries, women played a key role in making the intervention work, and the technique of fuzzy transitive closure allows examination of the impact of the intervention on intermediate outcomes among men and women [22]. Another article describes how the use of fish for biological vector control became part of the intervention in Mexico, and examines its effectiveness [23].

One aim of the trial was to measure whether the intervention reduced household costs. Two articles from Mexico indicate that the intervention was associated with a reduction both in the household use of and costs of chemical anti-mosquito products and in the direct and indirect costs of cases of dengue illness [24, 25]. The costs of the intervention itself are also important and the article by Ledogar et al. provides information about this [19].

A final article reflects on ethical issues that may arise when researchers deliberately cede control of interventions to communities themselves [26]. Such ethical considerations will be important as more researchers move towards community-led CRCTs, as proposed by Neil Andersson in his editorial in this supplement [27].

Typically, the articles that arise from a major trial appear in a variety of journals chosen by the authors according to the particular focus of each article. From a knowledge translation point of view, this can fragment the message and might mean that stakeholders miss out on parts of the story. Bringing together the articles in one place, such as this supplement, makes the whole canvas visible. It supports the use of the RCT as knowledge translation. Another knowledge translation effort related to the *Camino Verde* trial was the organisation of a symposium at a major dengue conference [28]. Pharmaceutical companies regularly use RCTs, now a legal requirement for new medications in many countries, in advertising to promote the use of their products. We need to get better at using RCTs as knowledge translation in the public health field.
